# Two-Year Overview of Theta-Burst Stimulation for Treatment-resistant Depression: Assessing Efficacy and Outcomes

**DOI:** 10.1192/j.eurpsy.2025.2119

**Published:** 2025-08-26

**Authors:** R. P. L. Andrade, C. P. Desport, C. Costa, C. Gomes, A. Dias, S. N. Martins, V. Covelo, P. Valente

**Affiliations:** 1 Serviço de Psiquiatria, ULS Viseu Dão-Lafões, Viseu; 2 Hospital de Magalhães Lemos, ULS de Santo António, Porto, Portugal

## Abstract

**Introduction:**

Major depressive disorder (MDD) is a very common and debilitating disorder. MDD accounts for 4.3% of the global burden of disease, is among the largest single causes of disability worldwide, and is an important cause of premature death. Depression expands its negative influence in all aspects of life, being estimate that 12 billion productive workdays are lost every year to depression and anxiety.

On top of that, non-response to first line pharmacological and psychotherapeutic treatments are substantial, with treatment-resistant depression (TRD) affecting approximately one third of these patients. These patients are thus candidates for non-invasive neuromodulation procedures such as repetitive transcranial magnetic stimulation (TMS), included in all major treatment guidelines.

**Objectives:**

With this work we intend to present a descriptive analysis of the efficacy of the intermittent theta burst TMS (iTBS) protocol in patients with TRD who underwent this treatment at Hospital de Magalhães Lemos, Porto, since July 2022.

**Methods:**

We conducted an analysis of sociodemographic characteristics of patients who underwent treatment with iTBS. The primary outcome was the Beck’s Depression Inventory (BDI) score difference between the first and last sessions. Secondary outcome included the Montgomery-Asberg Depression Rating Scale (MADRS) applied to a smaller cluster of patients.

**Results:**

Since July 2022, more than 50 cycles of iTBS treatment have been performed.

More than 60% of the TRD patients enrolled scored positive changes with the treatment, on BDI. Improvements exceeded non-response in both sexes, irrespective of disease duration, and in nearly all age groups – except for the single patient under 25 years old. Positive changes were also observed with the MADRS, with more than 70% of this cluster of TRD patients scoring positive changes, including the patient under 25 years old who score non-response with BDI.

iTBS was also applied to a small number of patients diagnosed with treatment-resistant bipolar major depression, in whom positive changes outweighed non-response.

All iTBS cycles were performed without major adverse effects being reported.

**Conclusions:**

TMs, represented here by the iTBS protocol, is safe and effective in improving depressive symptoms when first line treatments are not. The positive effects extend to patients diagnosed with BD, despite the small number of patients present in our patient pool.

Combined with the logistical ease of its use, not requiring general anaesthesia or induction of seizures like electroconvulsive therapy, TMS presents itself as an important alternative in the treatment of TRD.

**Disclosure of Interest:**

None Declared

